# Survival Rate of Cells Sent by a Low Mechanical Load Tube Pump: The “Ring Pump”

**DOI:** 10.3390/mi11040447

**Published:** 2020-04-23

**Authors:** Kaoru Uesugi, Keizo Nishiyama, Koki Hirai, Hiroaki Inoue, Yoichi Sakurai, Yoji Yamada, Takashi Taniguchi, Keisuke Morishima

**Affiliations:** 1 Department of Mechanical Engineering, Osaka University, 2-1 Yamada-oka, Suita, Osaka 565-0871, Japan; uesugi@live.mech.eng.osaka-u.ac.jp (K.U.); konomimachi@gmail.com (K.H.); 2 Global Center for Medical Engineering and Informatics, Osaka University, 2-1 Yamada-oka Suita, Osaka 565-0871, Japan; 3 Department of Mechanical Systems Engineering, Ibaraki University, 4-12-1 Nakanarusawacho, Hitachi, Ibaraki 316-8511, Japan; 4 Aquatech Co., Ltd., 2-1-13 Ono, Daito, Osaka 574-0042, Japan; nishiyama@parc.osaka-u.ac.jp (K.N.); inoue@ringpump-aquatech.co.jp (H.I.); sakurai@ringpump-aquatech.co.jp (Y.S.); yamada@ringpump-aquatech.co.jp (Y.Y.); taniguchi@ringpump-aquatech.co.jp (T.T.); 5 Phonics Center, Osaka University, 2-1 Yamada-oka Suita, Osaka 565-0871, Japan

**Keywords:** cell culture system, full-press ring pump, microfluidic system, mid-press ring pump, ring pump

## Abstract

A ring pump (RP) is a useful tool for microchannels and automated cell culturing. We have been developing RPs (a full-press ring pump, FRP; and a mid-press ring pump, MRP). However, damage to cells which were sent by the RP and the MRP was not investigated, and no other studies have compared the damage to cells between RPs and peristaltic pumps (PPs). Therefore, first, we evaluated the damage to cells that were sent by a small size FRP (s-FRP) and small size MRPs (s-MRPs; gap = 25 or 50 μm, respectively). “Small size” means that the s-FRP and the s-MRPs are suitable for microchannel-scale applications. The survival rate of cells sent by the s-MRPs was higher than those sent by the s-FRP, and less damage caused by the former. Second, we compared the survival rate of cells that were sent by a large size FRP (l-FRP), a large size MRP (l-MRP) (gap = 50 μm) and a PP. “Large size” means that the l-FRP and the l-MRP are suitable for automated cell culture system applications. We could not confirm any differences among the cell survival rates. On the other hand, when cells suspended in Dulbecco’s phosphate-buffered saline solution were circulated with the l-MRP (gap = 50 μm) and the PP, we confirmed a difference in cell survival rate, and less damage caused by the former.

## 1. Introduction

Various pumps have been considered for biotechnological utilizations. For example, a centrifugal pump, a peristaltic pump (PP), a diaphragm pump, a monoscrew pump, a rotary bane pump and an eccentric rotor pump were evaluated for the cell concentration process in [1]. Additionally, a centrifugal pump, a PP, a gravity-driven pump, a surface tension-based pump, an osmosis-based pump, a syringe pump, a vacuum pump and an electrokinetic pump were employed for microfluidic cell culturing using microfluidic devices [2].

Microfluidic platforms (microfluidic devices) have advantages of small size and high throughput experimentation [2]. Additionally, because microfluidic devices can control chemical and physical environments, these devices have been applied to small-scale cell cultivation systems such as lab-on-a-chip [2,3], organ- or body-on-a-chip [4,5] and a human-on-a-chip [6]. Other applications of microfluidic platforms include cell sorting systems [7,8], a microbioreactor [9], and microdroplet-based cell cultivation systems [10,11,12].

When injecting or transporting solutions such as cell suspensions, reagents or low viscosity fluids, various pump systems are used, and these pump systems are essential components in microfluidic platforms [2]. Although some studies have used syringes for supplying and introducing cell suspensions into a microchannel [5,11,12,13], syringes have some technical problems. First, they cannot supply solutions continuously. Syringes can only push out solutions that are stored in them, so they cannot continuously supply new solutions from outside. Exchanging an emptied syringe with a filled one can lead to contamination of the cultivation system. Second, syringes are large in size. On the other hand, solutions can be sent through a pump, so that a tube pump like the PP can send the solutions continuously from the outside. Because PPs do not store solutions in themselves, their size can be reduced [14]. By stroking their tubes with rollers, PPs can send solutions without changing the source of liquid and the PPs can send the solution in again. Based on these properties, PPs have been used for cultivation systems [15,16,17] and bioreactors [9,18,19].

**Figure 1:**
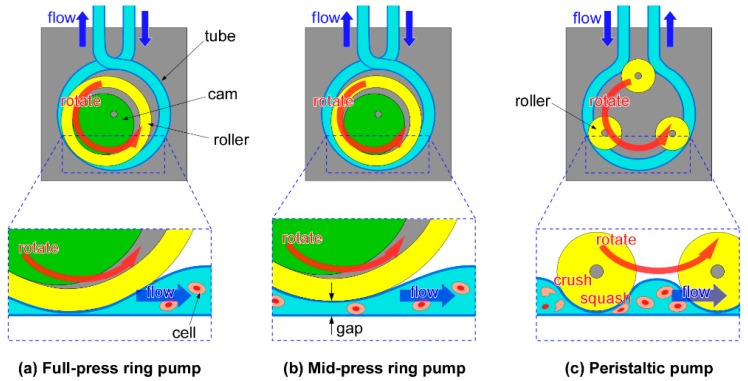
Schematic illustrations of three types of tube pumps. (**a**) The full-ring press pump (FRP) has a large size roller to stroke the tube, and the cells are not easily crushed. (**b**) The mid-press ring pump (MRP) also has a large size roller, but it keeps the gap between the inner wall of the tube. Therefore, the MRP is able to send the cell suspensions through the micro space (the gap). (**c**) The peristaltic pump (PP) has small rollers, and these rollers can easily crush cells.

However, when sending cell suspension solutions with PPs, cells can be crushed or squashed by the stroking motions of the tubes (Figure 1b) [20,21]. When cells are crushed, they undergo necrosis, or, if they do not die, the cells can experience mechanical effects. It has been reported that the characteristics of cells which were loaded with a mechanical stimulation were changed [22]. Excessive stroking of the PP can cause problems, including the tube becoming mechanically damaged and the tube is deforming, so that its contents are pushed out in the stroke direction by the rollers.

Therefore, we have proposed another type of tube pump, a “ring pump” (RP) for sending cell suspensions. The RP has one large-sized roller. Thus, the tube of the RP is pushed and stroked more gently compared to the tube of the PP. And because the tube is not stroked strongly, it will last longer and less damage may occur to cells in the suspensions. The RP can send solutions continuously and flow can be controlled from outside the tube in the same way as the PP. Moreover, since the structure of the RP is simple and has only a few parts, the space required for installation is smaller compared to other pump systems. There have been reports on using RPs for microfluidic devices [23,24]. Additionally, RPs which have been connected to microfluidic devices were also used for cell culturing [25,26]. Based on characteristics of the RP, we proposed a mid-press RP (MRP). The MRP can send solutions through a microscopic space (the gap between stroking points of the tube). As a matter of convenience, we call the standard type RP the full-press RP (FRP).

However, the effects on cells which are sent by the RP have not yet been reported, nor has the damage to cells sent by the FRP and the MRP been studied. To the best of our knowledge no comparisons for the damage to cells sent by the MRPs and PPs have been made.

Therefore, we investigated the damage to cells which were sent by the RPs. The characteristics of the RPs which were used in this study are summarized by Table 1. First, we studied the differences in damage to cells which were sent by the small-size full-press ring pump (s-FRP) and the small size mid-press ring pumps (s-MRPs) (gap = 25 or 50 μm). We consider “small size” to mean that the s-FRP and the s-MRP are suitable for applications on the scale of microchannels. The cell suspension was circulated in a flow circuit consisting of a silicon tube with the s-FRP and the s-MRP, and the survival rate was derived. Sent cells were harvested and seeded on a culture surface, and the proliferation of cells was observed. Then, we studied the differences in damage to cells which were sent by the large size full-press ring pump (l-FRP), large size mid-press ring pump (l-MRP) (gap = 50 μm) and the PP. In this study, we matched the size of the l-FRP and l-MRP to the size of a commercially available PP which has been used in an automated cell culture system. Thus, we consider “large size” to mean that the l-FRP and the l-MRP are suitable for applications on the scale of automated cell culture systems. Additionally, we applied cell suspensions which used a cell culture medium or Dulbecco’s phosphate-buffered saline (DPBS) solution with the l-MRP (gap = 50 μm) and the PP and derived the survival rates. By comparing these survival rates, we discussed the damage to cells which were sent with the l-FRP, the l-MRP (gap = 50 μm) and the PP.

## 2. Principle of the Ring Pump

Figure 1a illustrates the basic construction of the RP. By rotating an eccentric rotor (cam), a roller (ring) is rotated eccentrically. The tube, which is wound around the outer circumference of the roller, is pressed sequentially, and the liquid is sent through the tube. At the same time, the uncompressed tube expands due to the tube restoring force, and new liquid fills the tube. Because the roller can rotate freely with respect to the shaft, and grease is applied on the surface of the tube and the ring, friction between the ring and the tube is small. Furthermore, because solutions can be sent through the RP action, the RP can send solutions continuously from the outside, similar to the PP. The basic principle of the RP is the same as the PP (Figure 1c).

**Figure 2:**
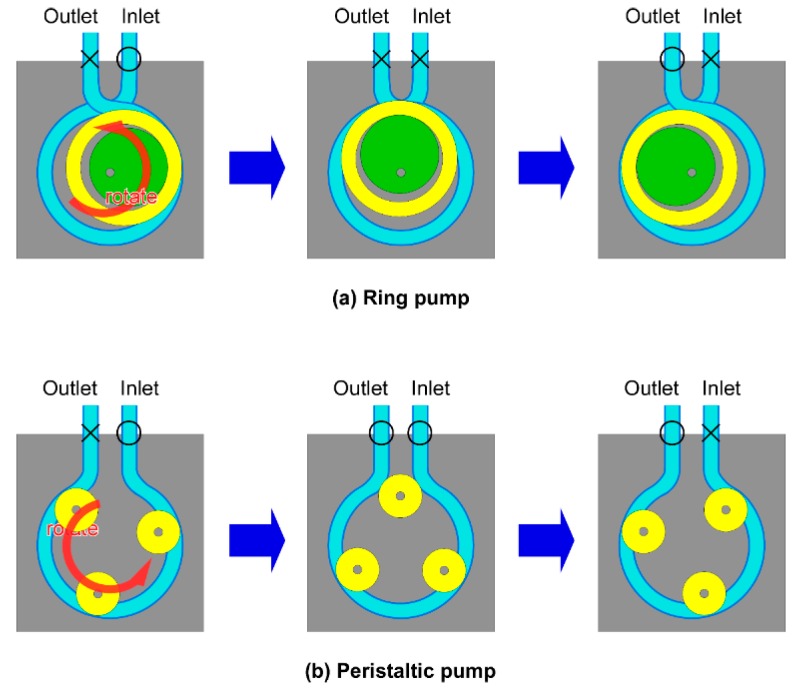
Schematic drawing showing prevention of backflow of liquid. (**a**) If the tube of the RP is crossed at the entrance and exit of the pump, there is no time interval when the inlet and the outlet are opened simultaneously. The yellow roller is always pressing one or the other openings closed. (**b**) If the tube of the PP is not crossed, there is a time interval when the inlet and the outlet are opened simultaneously (center image) and backflow can occur.

However, the RP differs from the PP in the following two points. First of all, the RP uses one roller which is relatively larger than rollers of the PP. If the size of the roller is larger, the extent of damage to cells becomes less [21]. Since the tube is gently compressed with the single large roller, it is considered that the space containing the cell suspension is large, and the cells cannot be crushed and squashed easily. On the other hand, because the PP squeezes the tube hard using multiple rollers, the cell suspension fills multiple small spaces from which it is sent. As a result, the cells are easily crushed and squashed or they may be loaded after being mechanically damaged. If the roller number is larger, the extent of damage to cells becomes more [20]. In addition, since the tube is squeezed hard with multiple rollers, the fatigue damage to the tube is large. Because the RP uses a single roller, there is a smaller number of pump parts, and it is possible to miniaturize the pump size and reduce the cost. Second, the tube of the RP is crossed at the entrance and exit of the pump and this prevents backflow of liquid. If the tube of the RP is crossed at the entrance and exit of the pump, there is no time interval when the inlet and the outlet are opened simultaneously (Figure 2a). On the other hand, If the tube of the PP is not crossed, there is a time interval when the inlet and the outlet are opened simultaneously (Figure 2b).

Moreover, the RP can send solutions through the micro space (the gap) which is made by adjusting the axis of rotation of the cam (Figure 1b). We call this pump a “mid-press ring pump (MRP)”. Because the MRP maintains a gap between the inner wall of the tube, this MRP is able to send suspensions which contain relatively large particles (diameters from several micrometers to several hundred micrometers). Although a method that lowers damage to cells by increasing gap size has been shown for the PP [20,21], our work is the first trial of a RP.

## 3. Materials and Methods

### 3.1. Preparation of Cell Suspension

In this study, mouse fibroblast cells, NIH-3T3 (3T3), were used. The 3T3 cells were cultured on a cell-culture-treated polystyrene dish (3020-100, AGC Techno Glass Co., Ltd., Shizuoka, Japan) in Dulbecco’s modified Eagle medium (D-MEM, high glucose, Nakalai Tesque, Kyoto, Japan) containing 10% fetal bovine serum (26140-079, Thermo Fisher Scientific K.K., Waltham, MA, USA) and 1% penicillin-streptomycin solution (P4333-100ML, Sigma–Aldrich Co. LLC., St. Louis, MO, USA). After confirming 80% confluence, cells were suspended in DPBS (Dulbecco’s phosphate-buffered saline, Nakalai Tesque) solution and detached from culturing dishes by incubating with TrypLE Express Enzyme (12604-021, Thermo Fisher Scientific K.K.) for 3 min. Then, the detached cells were centrifuged at 1 × 10^3^ rpm for 5 min (himac CT 4D, Hitachi, Ltd., Tokyo, Japan), and the cells were suspended in the culture medium. The cell density in the suspended solution was about 4 × 10^5^ cells/mL.

### 3.2. Comparison of Damage to Cells Sent with The Small Size Full-Press Pump and The Small Size Mid-Press Ring Pumps

**Figure 3:**
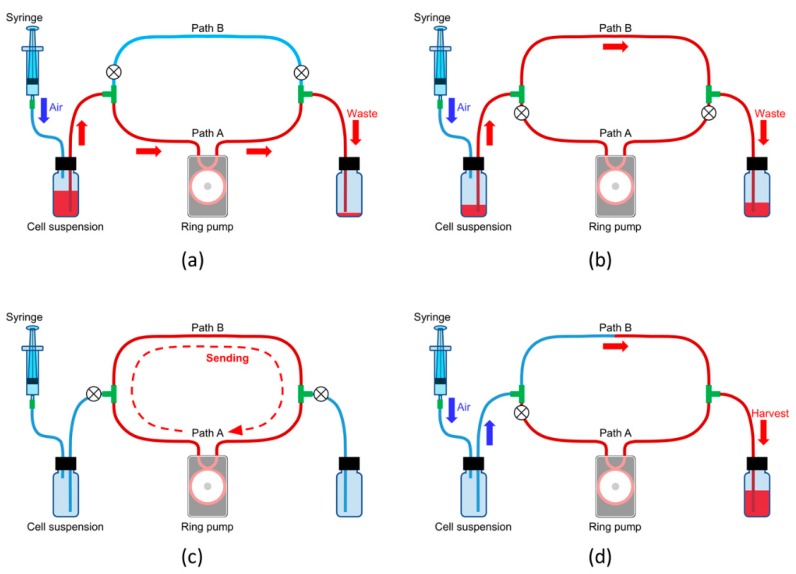
Schematic illustrations of the cell suspension sending system for the s-FRP and s-MRPs (gap = 25 or 50 μm). The system had two paths, path A and path B. Path A was integrated in the s-FRP or the s-MRP, and path B was used for the harvesting of suspended cells. (**a**) First, path A was filled with the suspension by air compression. (**b**) Second, path B was filled with the suspension. (**c**) After both paths were filled with the suspension, it was circulated by the ring pump. (**d**) Finally, cells of the circulated suspension were harvested by air pressure.

We confirmed damage to cells which were sent by a s-FRP (RP-HX01S-1A-DC3VS, Aquatech Co., Ltd., Osaka, Japan) and two s-MRPs (gap = 25 or 50 μm) (RP-HX01S-1A-DC3VS (altered), Aquatech Co., Ltd.) (Table 1 and Table 2). We consider “small size” to mean that the s-FRP and the s-MRP are suitable for applications on the scale of microchannels (Table 1). The inner diameter (ID) of the tubes which were embedded in the s-FRP and the s-MRP was 1 mm, and their outer diameter (OD) was 2 mm. In this experiment, since the size (volume) of the flow systems was smaller than that of other experiments (Section 3.4 and Section 3.6), the effect of loss of the cell suspension would be relatively large. Therefore, we constructed a special cell suspension sending system (Figure 3). The liquid circuit constructed of two paths, path A and path B. Path A was integrated into the RP. A silicon tube (ID, 1 mm and OD, 2 mm) was used for path A (length: 25 cm). Path B was used for harvesting the sample solution. A silicon tube (ID, 2 mm and OD, 3 mm) was used for path B (length: 10 cm). The total liquid volume of the two paths was about 510 μL.

The protocol for sending the cell suspension is explained below (Table 2). First, the cell suspension solution was filled into the tube of path A by air compression with a syringe when path B was stopped (Figure 3a). Then, path A was stopped, and solution flowed in path B (Figure 3b). After confirmation that the cell suspension solution filled the entire pathways, the cell suspension was circulated in them by the RP for about 19 min (Figure 3c). The experimental flow rate was 400 μL/min, the cells passed through the pump about 15 times, and the pump operating time was 19.1 min. To equalize the flow rate for each pump, the rotation frequencies of the RP were adjusted. Finally, the cells in the solution in path B were harvested by air compression (Figure 3d). In the control experiment, the cell suspension solution was left statically in path A and path B without sending the cell suspension through by pump action. After about 19 min, cells were harvested from path B by air compression. To avoid contamination during cell incubation, the tubes were sterilized using an autoclave before experiments. Additionally, the tubes were washed before each experiment using ethanol and DPBS solution, and all manipulations were carried out on a clean bench.

After harvesting of cells, for recognition of dead cells, staining was done with trypan blue (T10282, Life Technologies Co., CA, USA). Then, cells were counted with a cell counter (Countess II, FL, Thermo Fisher Scientific K.K.). A ratio was derived as the number of live cells divided by the number of total cells (the sum of the numbers of live cells and dead cells). Then, the ratio of live cells sent by the pump was divided by the ratio of live cells of control, and that was multiplied by 100 to obtain the survival rate of cells. The experiment was carried out at room temperature (24 °C) and repeated three times for each RP; the weighted average and standard error were calculated.

### 3.3. Cell Culture and Observations

Cells sent by the s-FRP and the s-MRPs (gap = 25 or 50 μm) were seeded on separate 48-well plates (353078, Corning Inc., New York, NY, USA). The volume of cell suspension solution which was dispensed to a well was 50 μL. The conditions for cell culturing were the same as for the experiment of Section 3.1. Cells were incubated for 2 days and observed with a phase-contrast microscope (BZ-X710, Keyence, Osaka, Japan).

### 3.4. Comparison of Damage of Cells Sent with the Large Size Full-Press Ring Pump, Large Size Mid-Press Ring Pump and the Peristaltic Pump

**Figure 4:**
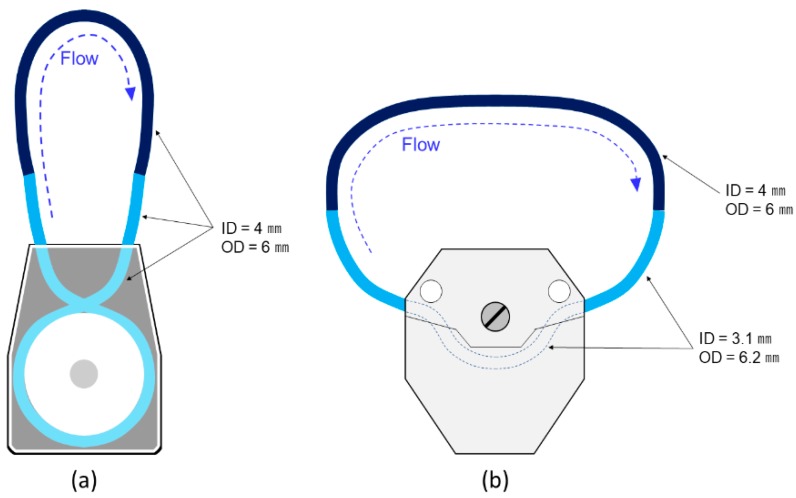
Schematic illustrations of the cell suspension sending systems for the l-FRP, the l-MRP and the PP. The circulation paths of the systems were structured with silicon tubes. (**a**) The silicon tubes used for the l-FRP and l-MRP had ID of 4 mm and OD of 6 mm. The total length of the two paths was 30 cm and the total liquid volume was about 3.8 mL. (**b**) The silicon tubes used for the peristaltic pump had ID of 4 mm and OD of 6 mm (length: 15 cm), and ID of 3.1 mm and OD of 6.2 mm (length: 30.5 cm). The total liquid volume of the two paths was about 4.2 mL.

We confirmed damage to cells which were sent by a l-FRP (RP-M04S-50Z-DC24V, Aquatech Co., Ltd.), a l-MRP (gap = 25 or 50 μm) (RP-M04S-50Z-DC24V (altered), Aquatech Co., Ltd.) and a PP (Easy-Load 07516-10, Yamato Scientific Co., Ltd., Tokyo, Japan) (Table 1 and Table 2). This PP has been used in automated cell culture systems (e.g., CELLAFORTE, Nipro Co., Osaka, Japan). We consider “large size” to mean that the l-FRP and the l-MRP are suitable for applications on the scale of automated cell culture systems (Table 1). The tube sizes of the l-FRP and the l-MRP (ID = 4 mm, OD = 6 mm) matched those of the PP (ID = 3.1 mm, OD = 6.2 mm). Thus, the volume of solutions was larger, and there was no need to construct a more accurate experimental set up. Figure 4 is a schematic illustration of the circulation flow systems for the l-FRP, l-MRP and the PP. The silicon tubes which were used for the l-FRP and the l-MRP circulation flow system had ID of 4 mm and OD of 6 mm (Figure 4a). The total length of the two paths was 30 cm, and the total liquid volume was about 3.8 mL. The silicon tubes which were used for the PP had ID of 4 mm and OD of 6 mm (length: 15 cm) and ID of 3.1 mm and OD of 6.2 mm (length: 30.5 cm). The total liquid volume of these two paths was about 4.2 mL. The suspension was filled into the tubes with a 5 mL pipette. After confirming solution completely filled the pathways, the cell suspension was circulated within them by the l-FRP, l-MRP or PP for about 5 min. The experimental flow rate was 30 mL/min, the cells passed through the pump about 39 times (l-FRP, l-MRP) and 36 (PP) times, and the pump operating time was 300 s. In order to equalize the flow rate for each pump, the rotation frequencies of each pump were adjusted. In the control experiment, the cell suspension solution was left statically in the silicon tube (ID = 4 mm; OD = 6 mm; total volume = 5 mL) for 5 min without sending it through. Finally, the cells in the suspension solution were harvested by air compression, and live cells were counted by the cell counter (Countess II, FL, Thermo Fisher Scientific K.K.). The survival rate of cells was derived by the same method as in the Section 3.2 experiment. The experiment was carried out at room temperature (24 °C) and repeated two times for each pump; the average and standard error were calculated.

### 3.5. Cell Culture and Observations

The 3T3 cells were cultured and detached from the culture dish (Section 3.1). Then, the detached cells were centrifuged (1 × 10^3^ rpm, 5 min), and the cells were suspended in DPBS and left for 4 h at room temperature (24 °C). The cell density in the suspended solution was about 4 × 10^5^ cells/mL.

### 3.6. Comparison of Damage of Cells Suspended in The DPBS Solution Sent with the Large Size Full-Press Ring Pump, the Large Size Mid-Press Ring Pump and the Peristaltic Pump

We confirmed damage to cells of the sent suspension in DPBS solution when using the l-MRP (gap: 50 µm) (RP-M04S-50Z-DC24V (altered), Aquatech Co., Ltd.) and the PP (Easy-Load 07516-10, Yamato Scientific Co.) (Table 1 and Table 2). The circulation flow systems were the same as those of the Section 3.5 experiment.

The cell suspension, which was suspended in DPBS solution was introduced into the silicon tube, then, it was sent by the RP or the PP. In both cases, the flow rate was 100 mL/min, the cells passed through the pump about 145 times (l-MRP) and 131 times (PP), and the pump operating time was 330 s. In the control experiment, the cell suspension solution was left in the silicon tube (ID = 4 mm; OD = 6 mm; total volume = 5 mL) for 5.5 min without sending it through. Finally, the cells in the solution were harvested by air compression, and live cells were counted by the cell counter (Countess II, FL, Thermo Fisher Scientific K.K.). The survival rate of cells was derived by the same method as in the Section 3.2 experiment. The experiment was carried out at room temperature (24 °C), one time for each pump, and the average and standard error were calculated.

## 4. Results

### 4.1. Comparison of Damage of Cells Sent with the Small Size Full-Press Ring Pump and the Small Size Mid-Press Ring Pumps

**Figure 5:**
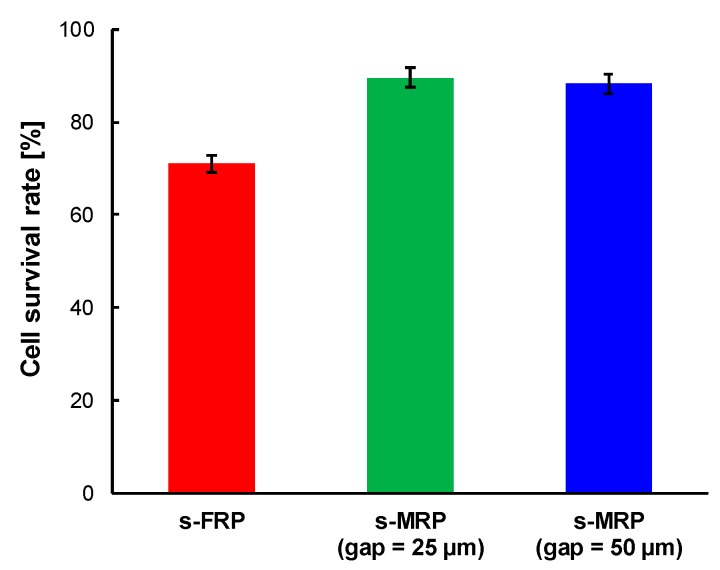
Survival rate of cells which were sent with the s-FRP and two s-MRPs. The survival rates were 71% ± 2% (s-FRP), 90% ± 2% (s-MRP; gap = 25 µm) and 88% ± 2% (s-MRP; gap = 50 µm). The survival rate was derived by the weighted average, and the experiment was carried out three times for each pump.

Figure 5 shows the survival rate of cells which were sent with the s-FRP and s-MRPs. The ratio of the number of live cells to the number of total cells was 9.4 ± 0.1 (control), 6.7 ± 0.3 (s-FRP), 8.4 ± 0.3 (m-MRP, gap = 25 µm) and 8.3 ± 0.3 (s-MRP; gap = 50 µm). The survival rates were 71% ± 2% (s-FRP), 90% ± 2% (s-MRP; gap = 25 µm) and 88% ± 2% (s-MRP; gap = 50 µm).

**Figure 6:**
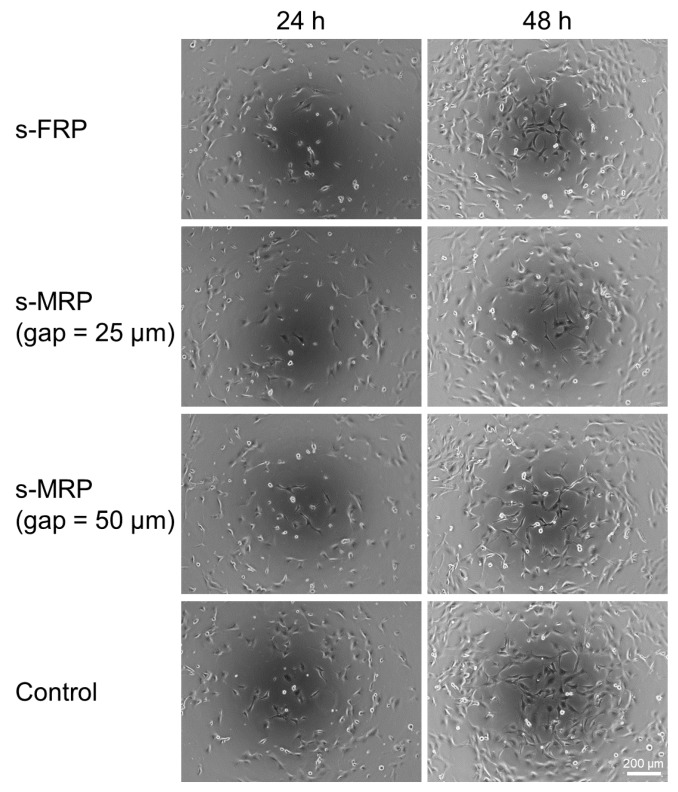
Phase-contrast microscopy images of cultured cells which were sent with the s-FRP and s-MRPs (gap = 25 or 50 μm). All cells which were sent using each of the three RP were adhered on the cell culture polystyrene surface and proliferated. The control cells were also adhered on the cell culture polystyrene surface and proliferated. Scale bar: 200 µm.

Figure 6 shows the phase-contrast microscope images of cultured cells which were sent with the s-FRP and s-MRPs. All cells sent with each RP were adhered on the cell culture polystyrene surface and proliferated. The control cells were also adhered on the cell culture polystyrene surface and proliferated.

### 4.2. Comparison of Damage of Cells Sent with the Large Size Full-Press Ring Pump, Small Size Mid-Press Ring Pump and the Peristaltic Pump

**Figure 7:**
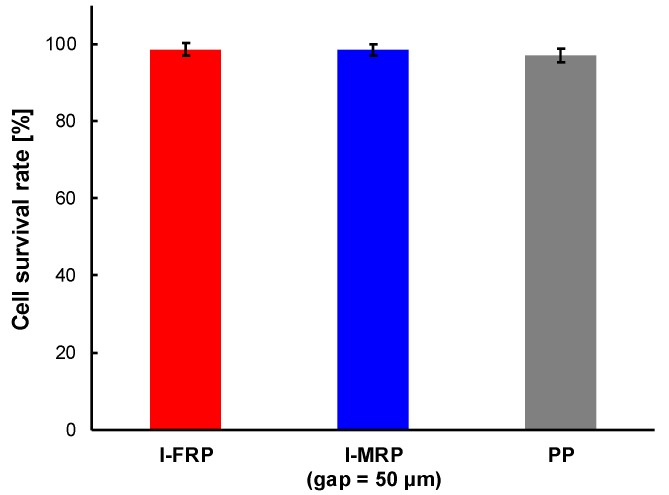
Survival rate of cells which were sent with the l-FRP and the l-MRP (gap = 50 μm) and the PP. The survival rates were 99% ± 2% (l-FRP), 98% ± 2% (l-MRP; gap = 50 µm) and 97% ± 2% (PP). The experiment was carried out two times for each pump.

Figure 7 shows the survival rate of cells which were sent with the l-FRP and the l-MRP and the PP. The ratio of the number of live cells to the number of total cells was 9.8 ± 0.1 (control), 9.6 ± 0.2 (l-FRP), 9.6 ± 0.2 (l-MRP, gap = 50 µm) and 9.5 ± 0.2 (PP). The survival rates were 99% ± 2% (l-FRP), 98% ± 2% (l-MRP; gap = 50 µm) and 97% ± 2% (PP).

### 4.3. Comparison of Damage of Cells Suspended in DPBS Solution Sent with the Large Size Full-Press Ring Pump, the Large Size Mid-Press Ring Pump and the Peristaltic Pump

**Figure 8:**
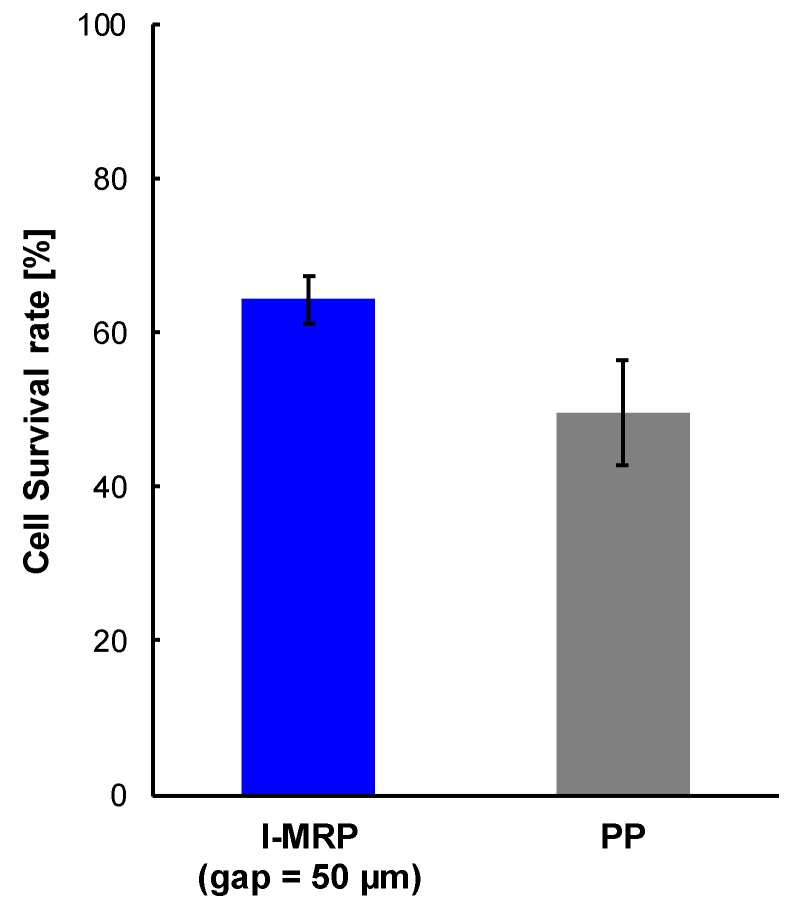
Survival rate of cells which were sent with the l-MRP (gap = 50 µm) and the PP. The survival rates were 64% ± 6% (l-MRP) and 50% ± 7% (PP). The experiment was carried out one time for each pump.

Figure 8 shows the survival rate of cells which were sent with the l-MRP (gap = 50 µm) and the PP. The ratio of the number of live cells to the number of total cells was 7.5 ± 0.2 (control), 4.9 ± 0.4 (l-MRP; gap = 50 µm) and 3.7 ± 1 (PP). The survival rates were 64% ± 6% (l-MRP; gap = 50 µm) and 50% ± 7% (PP).

## 5. Discussion

The RP is expected to function well as a pump for sending cell suspensions into microchannels or automated cell culture systems. Using the RP ensures that cells are not loaded with any mechanical damage. This is especially important for the MRP, which has more spaces inside the pressed tube through which the cell suspension can be sent with just slight damage. However, there have been few studies which researched the effects of RPs. Therefore, we studied the damage to cells when sent by RPs.

In the first experiment, we used the s-FRP and s-MRP. The survival rate of cells which were sent by the s-FRP (71% ± 2%) was lower than that of the s-MRPs (gap 25 µm, 90% ± 2%; gap 50 µm, 88% ± 2%) (Figure 5). This indicates that the cells had experienced mechanical stress due to the peristaltic motion of the tube which was used for the s-FRP. It was reported that the gap reduced the damage to cells in the PP [20,21]. Therefore, this result is reasonable. Furthermore, it was also reported that the damage to cells was reduced with the increment of gap size [21]. On the other hand, there was almost no difference between the survival rates of the pumps-MRP having a gap of 25 µm or 50 µm. The diameters of floating 3T3 cells are around 10 µm, and this size is sufficiently smaller than the gap of the s-MRPs. Thus, there would likely be few possibilities that cells were damaged by pressing of the tube for the s-MRPs of both gap sizes. These results indicate favorable conditions for using the s-FRP and the s-MRPs for sending cell suspensions. If the sending pressure and sending speed have priority, the s-FRP is suitable. On the other hand, if the survival rate of cells has priority, the s-MRP is suitable.

The cells which were sent by the s-FRP and the s-MRPs were cultured and observed with a phase-contrast microscope (Figure 6). In this experiment, we could not confirm any difference between cells sent with the s-FRP and s-MRPs. We thought that any difference would be canceled by variations in the cell suspension density, the cell seeding density or the conditions of cell propagation. While we could not confirm any difference in culturing, we saw that damage to cells which were sent by the s-FRP and s-MRPs was not significantly different from that of the control. The control cells were also adhered on the cell culture polystyrene surface and proliferated. This means that the cells which were sent by the s-FRP and s-MRPs were loaded with only slight damage which did not affect cell proliferation.

**Figure 9:**
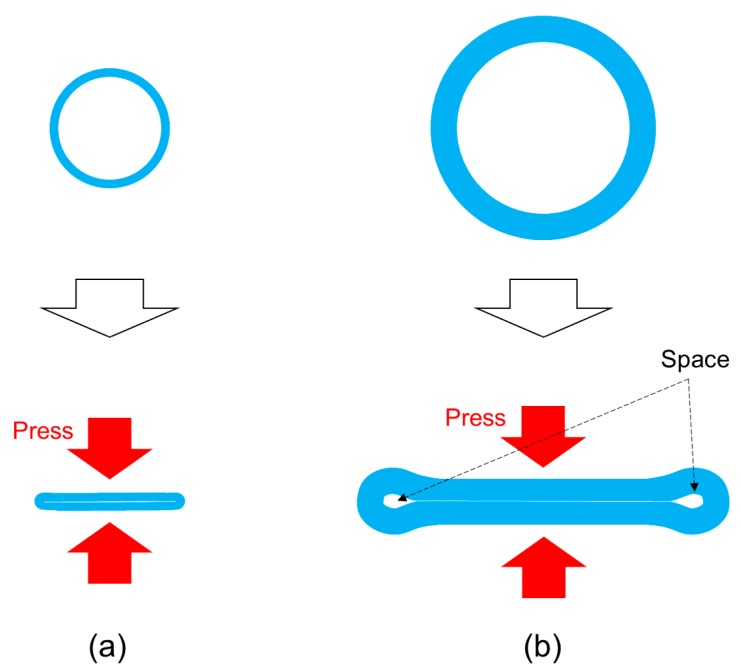
Schematic illustrations showing the shapes of the collapsed tubes. (**a**) When the small diameter tube which was used for the s-FRP and the s-MRP was loaded with a certain pressing force by the roller, the tube became completely flat, and there was no space for solution flow. (**b**) When the large diameter tube was loaded with a certain pressing force, there were some spaces for solution flow with the l-MRP and the PP.

Figure 7 shows the survival rate of cells which were sent by the l-FRP, l-MRP and PP. The size of the l-FRP, l-MRP and the PP was the same and larger than the size of the s-FRP and the s-MRPs. There was almost no difference between the survival rates for all types of RPs (l-FRP, 99% ± 2%; l-MRP, gap = 50 µm, 98% ± 2%). This result differed from the results of the s-FRP and s-MRPs. Additionally, the survival rate of cells which were sent by the PP was almost the same value (97% ± 2%). Although the number of passes through the pump (36 times) and sending speed (30 mL/min) of the l-FRP, the l-MRP and the PP were larger than those of s-FRP and s-MRPs (15 times, 400 µL), there was little difference in the survival rate. The cell damage caused by pressing of the tube became larger with the increase of the sending speed and the number of passes through the pump. The reason for the similar survival rate may be due to the tube deformation. When the small tube which was used for the s-FRP was loaded with pressing force by the roller, the tube collapsed and became completely flat, and there were no spaces (Figure 9a). On the other hand, when the large tube which was used for the l-FRP and the PP was loaded with the pressing force, there were some spaces (Figure 9b). Even if pressing force is loaded to cells through the tube, cells go through the spaces. Therefore, it can be considered that there were no differences in the cell survival rates for the l-FRP, l-MRP and the PP.

Then, we sent cells which were suspended in DPBS using the l-MRP gap = 50 µm) and the PP. The respective survival rates were 64% ± 3% and 50% ± 7% (Figure 8). Differing from “experiment 2” (Table 2), we confirmed there was a difference between the survival rate of cells which were sent by the l-MRP and the PP. We considered there were two reasons related to this result. First, because cells were suspended in the DPBS solution for 4 h, cells were loaded with some initial damage. Second, because the number of passes through the pump and the sending speed of cells which were suspended in the DPBS solution (125 times, 100 mL/min) were larger than those of cells without DPBS treatment (15 times, 400 µL), cells had more damage. Therefore, the damage of cells was increased, and even light mechanical stimulation could affect them. The number of times that cells passed through the l-MRP (145) was larger than that of the PP (131); as the number of times that cells pass through the pump is increased, cells are damaged more. However, the cell survival rate of cells which were sent by the PP was lower than that of the l-MRP. Thus, when it is necessary to send suspensions with low cell damage, it is considered that the l-MRP is more suitable than the PP.

We considered reasons why the survival rate of cells that were sent by the PP was lower than that of the l-MRP from the viewpoint of fluid dynamics. In the PP, the shear stress and the pressure of liquid are significantly changed depending on the position (the position of the contracting part or the dilating part in the tube) [27,28]. Additionally, because the PP encloses the flow path (tube), the flow is trapped and refluxed, and liquid is mixed [29,30]. Changes of the shear stress and the pressure are also caused by this mixing. Because the number of rollers of the PP was more than that of the RP (in this study, the former had three and the latter had one), the frequency of loading by contraction-dilating and enclosing was also more than in the RP. This was based on the fact that the PP system had more areas where shear stress and pressure were changing than the RP had. Therefore, we assumed that the PP had many areas where cells were loaded with more mechanical damage than the RP was loaded with. Because cells are loaded with damage by shear stress [1,28], we considered that the damage to cells by the PP, which had more areas where the shear stress and pressure were changing, was larger than the damage to cells by the RP.

The MRP may be used not only with microchannels but also for dispensing and sending cell suspensions in automated cell culture systems, mass cultivation systems and freeze-preservation systems. The l-MRP can realize low-cost cultivation by decreasing damage to cells. By getting a sufficient gap size for the l-MRP, microspheres which are constructed from induced pluripotent stem cells (iPSCs) [31] and the microcarrier on which those iPSCs adhere [32] can also be sent.

The trend seen in the experiment using the l-MRP and the PP can be applied to the s-MRPs. Thus, when cell suspensions are sent into microfluidic devices, s-MRPs may be more suitable than small size PPs.

## 6. Conclusions

When introducing cell suspensions into microchannels or automated cell culture systems, RPs are a useful tool. However, there have been no studies which evaluated the damage to the cells which are sent with them. Therefore, we evaluated the damage to cells which were sent by RPs.

First, we evaluated the damage to cells which were sent with the s-FRP and the s-MRPs. The s-MRP have a small gap at the compressed part of the tube. The cell suspension was circulated through silicon tubes. We found the cell survival rate of the s-MRPs (gap = 25 or 50 μm) was higher than that of the s-FRP. Thus, the extent of damage to cells which were sent with the s-MRPs was less than that of the s-FRP.

Additionally, using the l-FRP and l-MRP (gap = 50 μm), we compared the RPs and the PP. We compared the survival rate of cells which were circulated in silicon tubes with the l-FRP, the l-MRP and the PP. There were no differences among their survival rates. The reason for this might be due to the spaces which were formed by the tube deformation. Even when pressing force was loaded to the tube, and the tube collapsed, the cells could pass through the spaces.

On the other hand, when cells which were suspended in DPBS solutions were circulated by the l-MRP (gap = 50 μm) and the PP, the survival rate of cells which were circulated with the l-MRP was higher than that of the PP. The damage to cells which were sent with the l-MRP was less than that of the PP.

The MRP can send solutions which contain suspended particles and little damage is caused. Thus, the MRP can be used not only for microfluidic systems but also for dispensing and sending of cell suspensions of automated cell culture systems, mass cultivation systems and freeze-preservation systems.

In this study, we wanted to validate the effects of the pump only. If the microchannel were connected to the pump system, validation would be influenced by various other factors (e.g., shear stress around the microchannel or the connection between the pump and the microchannel). Thus, we did not validate the effects of the pump system which consisted of the pump and the microchannel. However, it is also important to evaluate the effects of the pump system including the microchannel, and we will try to validate these effects in a future work.

## Figures and Tables

**Table 1:** Correspondence table showing names of pumps. Abbreviations: OD–outer diameter; ID–inner diameter.

Commercial Model Name	Common Name	Abbreviated Name	Gap Size (µm)	Pump Size w/h/d (mm)	Diameter of Roller (mm)	Tube Size and Length (mm), (cm)	Application
RP-HX01S-1A-DC3VS	small size full-press ring pump	s-FRP	0	20/32/22.2	12.83	ID = 1, OD = 2, length: 25 ID = 2, OD = 3, length: 10	microchannels
RP-HX01S-1A-DC3VS (altered)	small size mid-press ring pump	s-MRP	25	20/32/22.2			
RP-HX01S-1A-DC3VS (altered)	small size mid-press ring pump	s-MRP	50	20/32/22.2			
RP-M04S-50Z-DC24V (altered)	large size mid-press ring pump	l-MRP	0	74/74.5/24.8	42.7	ID = 4, OD = 6, length: 30	cell cultivation systems
RP-M04S-50Z-DC24V (altered)	large size mid-press ring pump	l-MRP	50	74/74.5/24.8			
Easy-Load 07516-10	peristaltic pump	PP	0	116/102/56	14.25 (measured value)	ID = 3.1, OD = 6.2, length: 30.5 ID = 4, OD = 6, length: 15	

**Table 2:** Correspondence table between pumps and experimental conditions.

Experiment Number	Section	Type of Pump	Commercial Model of Pump	Gap Size (µm)	Solution	Volume of Setup (Ml)	Flow Rate (mL/min)	Number of Passages of Cells Which Were Passed Through Pump	Running Time (min)	Cell Density of Suspension (cells/mL)
1	3.1 3.2 3.3 4.1	s-FRP	RP-HX01S-1A-DC3VS	0	Culture medium	0.51	0.4	about 15	19.1	4 × 10^5^
		s-MRP	RP-HX01S-1A-DC3VS (altered)	25						
		s-MRP		50						
		control	-	-			-	-		
2	3.4 4.2	l-FRP	RP-M04S-50Z-DC24V	0	Culture medium	3.8	30	about 39	5	4 × 10^5^
		l-MRP	RP-M04S-50Z-DC24V (altered)	50						
		PP	Easy-Load 07516-10	0		4.2		about 36		
		control	-	-		5	-	-		
3	3.5 4.3	l-MRP	RP-M04S-50Z-DC24V (altered)	50	DPBS	3.8	100	about 145	5.5	4 × 10^5^
		PP	Easy-Load 07516-10	0		4.2		about 131		
		control	-	-		5	-	-		
